# Endothelin-1 Mediates Brain Microvascular Dysfunction Leading to Long-Term Cognitive Impairment in a Model of Experimental Cerebral Malaria

**DOI:** 10.1371/journal.ppat.1005477

**Published:** 2016-03-31

**Authors:** Brandi D. Freeman, Yuri C. Martins, Oscar B. Akide-Ndunge, Fernando P. Bruno, Hua Wang, Herbert B. Tanowitz, David C. Spray, Mahalia S. Desruisseaux

**Affiliations:** 1 Department of Pathology, Albert Einstein College of Medicine, Bronx, New York, United States of America; 2 Department of Biochemistry, Albert Einstein College of Medicine, Bronx, New York, United States of America; 3 Department of Medicine, Albert Einstein College of Medicine, Bronx, New York, United States of America; 4 Dominick P. Purpura Department of Neuroscience, Albert Einstein College of Medicine, Bronx, New York, United States of America; Michigan State University, UNITED STATES

## Abstract

*Plasmodium falciparum* infection causes a wide spectrum of diseases, including cerebral malaria, a potentially life-threatening encephalopathy. Vasculopathy is thought to contribute to cerebral malaria pathogenesis. The vasoactive compound endothelin-1, a key participant in many inflammatory processes, likely mediates vascular and cognitive dysfunctions in cerebral malaria. We previously demonstrated that C57BL6 mice infected with *P*. *berghei* ANKA, our fatal experimental cerebral malaria model, sustained memory loss. Herein, we demonstrate that an endothelin type A receptor (ET_A_) antagonist prevented experimental cerebral malaria-induced neurocognitive impairments and improved survival. ET_A_ antagonism prevented blood-brain barrier disruption and cerebral vasoconstriction during experimental cerebral malaria, and reduced brain endothelial activation, diminishing brain microvascular congestion. Furthermore, exogenous endothelin-1 administration to *P*. *berghei* NK65-infected mice, a model generally regarded as a non-cerebral malaria negative control for *P*. *berghei* ANKA infection, led to experimental cerebral malaria-like memory deficits. Our data indicate that endothelin-1 is critical in the development of cerebrovascular and cognitive impairments with experimental cerebral malaria. This vasoactive peptide may thus serve as a potential target for adjunctive therapy in the management of cerebral malaria.

## Author Summary

The parasite *Plasmodium falciparum* is the primary cause of cerebral malaria, a neurological manifestation of severe malaria. Cerebral malaria results in disturbances to the blood vessels of the brain, eventually leading to damage to the blood-brain barrier. This damage can lead to adverse, debilitating neurological complications, particularly in children and individuals with compromised immune systems. Yet there is still a considerable gap in understanding the causes of the detrimental neurological effects of *P*. *falciparum* infection. We employed a multidisciplinary approach to delineate the mechanisms by which *Plasmodium* infection causes these abnormalities. The vasoactive peptide endothelin-1 is implicated in a variety of neurological and inflammatory diseases. Using mouse experimental models of cerebral malaria, we demonstrated that targeting this protein resulted in stabilization of the blood vessels in the brain, decreased the influx of inflammatory cells to the brain vessels, and preserved the integrity of the blood-brain barrier, eventually leading to improved cognitive function and improved survival rates in mice with infection. It is our hope that our work will help extend understanding of the causes of cerebral malaria in humans, and may eventually lead to therapies for preservation or salvaging of neurological function in the management of this disease.

## Introduction

Malaria, caused by infection with the intraerythrocytic parasite *Plasmodium falciparum*, is a potentially life threatening disease, with significant morbidity and devastating economic consequences in developing countries [1]. Acute infection causes a spectrum of disease including cerebral malaria (CM), the most severe manifestation of infection. Despite extensive research, there remains a critical gap in our understanding of the mechanisms that promote the CNS pathology, and the ensuing neurocognitive deficits that result from infection. Consequently, the case fatality from CM remains high at 20%, and one in every four survivors develops long-term neurological sequelae even after successful parasite eradication [2–4].

Experimental CM (ECM) models exhibit several pathological alterations observed in human CM, including cerebral vascular obstruction, vasoconstriction, reduced cerebral blood flow (CBF), brain hemorrhage, BBB disruption, inflammation, and neurological impairment [5–11]. Such models enable researchers to examine the cellular and molecular mechanisms involved in CM pathology. Our previous data suggest that endothelin-1 (ET-1), a potent vasoactive peptide, is increased during ECM and may contribute to the vasculopathy observed during malarial infection [6, 12]. Although normally expressed in the CNS of healthy individuals, ET-1 levels increase in response to stress and have been implicated in a broad array of conditions, including stroke and subarachnoid hemorrhage [6, 13–15]. ET-1 is also associated with inflammation, microglial activation, BBB breakdown, and likely contributes to the neuroinflammatory process and ensuing neurocognitive impairment observed in CM [10, 13–16].

The actions of ET-1 are mediated through two G-protein-coupled receptors ET_A_ and ET_B_, which modulate inflammation, BBB integrity, and vascular tone [13–16]. Our published data demonstrate elevated mRNA expression of ET-1 and of the ET receptors in the brains of mice with ECM, in association with reduced CBF [6]. These reports mirror observation in patients with severe *P*. *falciparum* malaria [17, 18].

We recently reported that ET_A_ receptor antagonism reduced the incidence of brain hemorrhage in ECM [12]. Using a multidisciplinary approach, we have now extended these studies to demonstrate, for the first time, a mechanistic role for ET-1 in the development of endothelial dysfunction and the generation of ECM. Herein we report that ET-1 is essential in mediating the vascular dysfunction and the subsequent associated cognitive impairment during ECM. In this regard, we demonstrate that ET_A_ receptor antagonism prevents visual memory impairments as a consequence of attenuating cerebral vasoconstriction, BBB disruption, and vascular congestion.

## Results

### ET_A_ receptor antagonism improves survival and disease severity during the course of acute P. berghei ANKA (PbA)-infection

**Fig 1:**
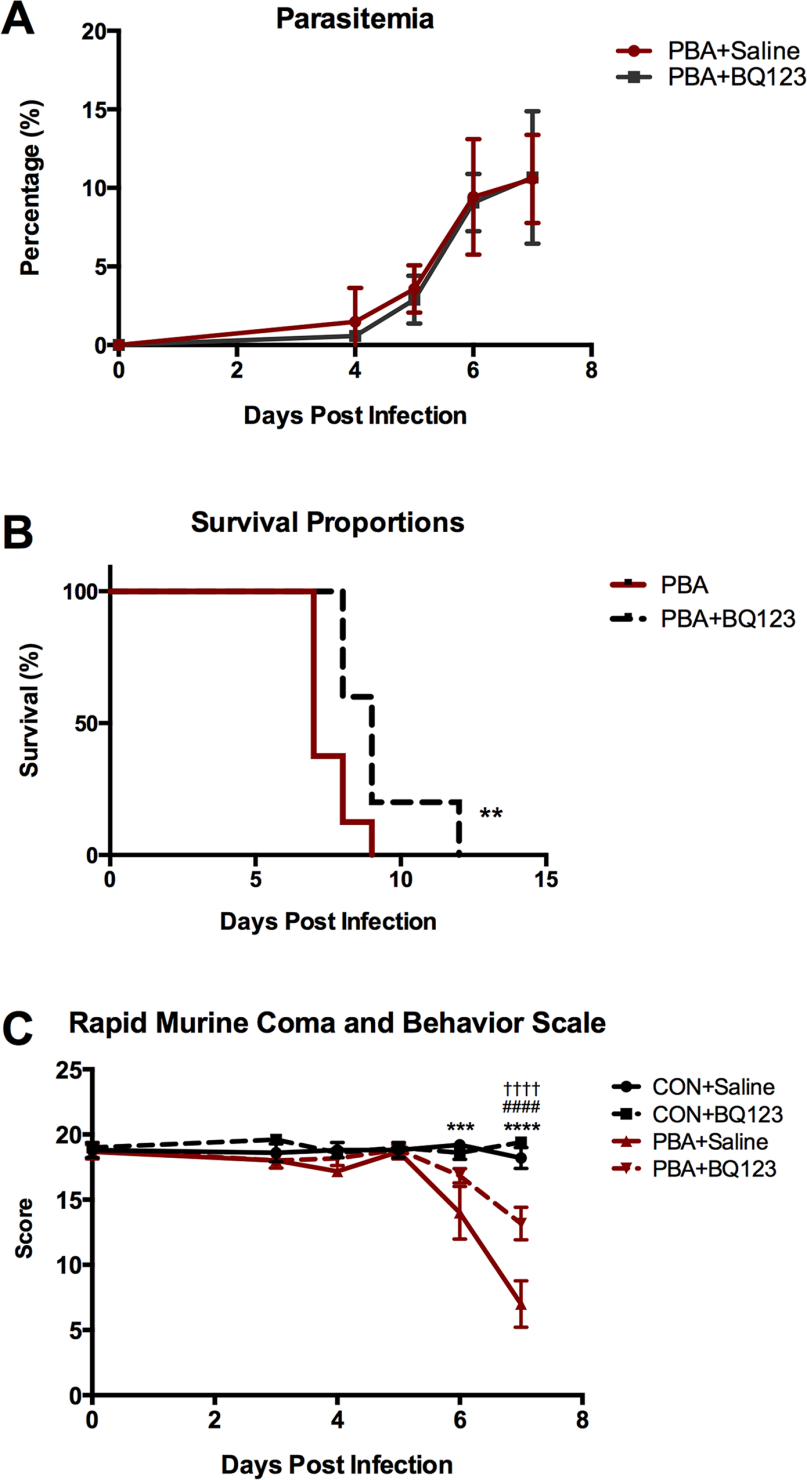
BQ123 effects on parasitemia, survival, and disease severity. **(A)** BQ123 treatment had no effect on parasitemia in PbA-infected mice; Two-way ANOVA. **(B)** BQ123 treatment significantly prolonged survival in PbA-infected mice; Log-rank (Mantel-Cox) test. **(C)** PbA-infected mice demonstrated gradual decreases in the rapid murine coma and behavior scale (RMCBS) after infection with significant changes occurring at 6 and 7dpi. Although BQ123-treated infected mice also scored significantly lower in the RMCBS than uninfected controls at 7dpi, BQ123 significantly dampened the decline in RMCBS scores in infected mice; Two-way ANOVA; ** = p < 0.01, *** = p < 0.001 and **** p < 0.0001. **For graph C**: * = Con vs. PbA; # = Con vs. PbA+BQ123; † = PbA vs. PbA+BQ123. n = 10/group.

We examined the effect of the selective ETA receptor antagonist, BQ123, on uninfected and PbA-infected mice. Despite having no antimalarial properties (Fig 1A), BQ123 treatment significantly prolonged survival following PbA infection (Fig 1B). BQ123 also improved disease severity and the development of neurological signs associated with ECM, in PbA-infected mice, as measured by the rapid murine coma and behavior scale (RMCBS) (Fig 1C). Interestingly, BQ123 had no effect on weight or on body temperature in healthy uninfected mice, nor on the body weight or temperature fluctuations in PbA-infected mice (S1 Fig).

### ET_A_ receptor antagonism prevents memory dysfunction during acute PbA-infection

**Fig 2:**
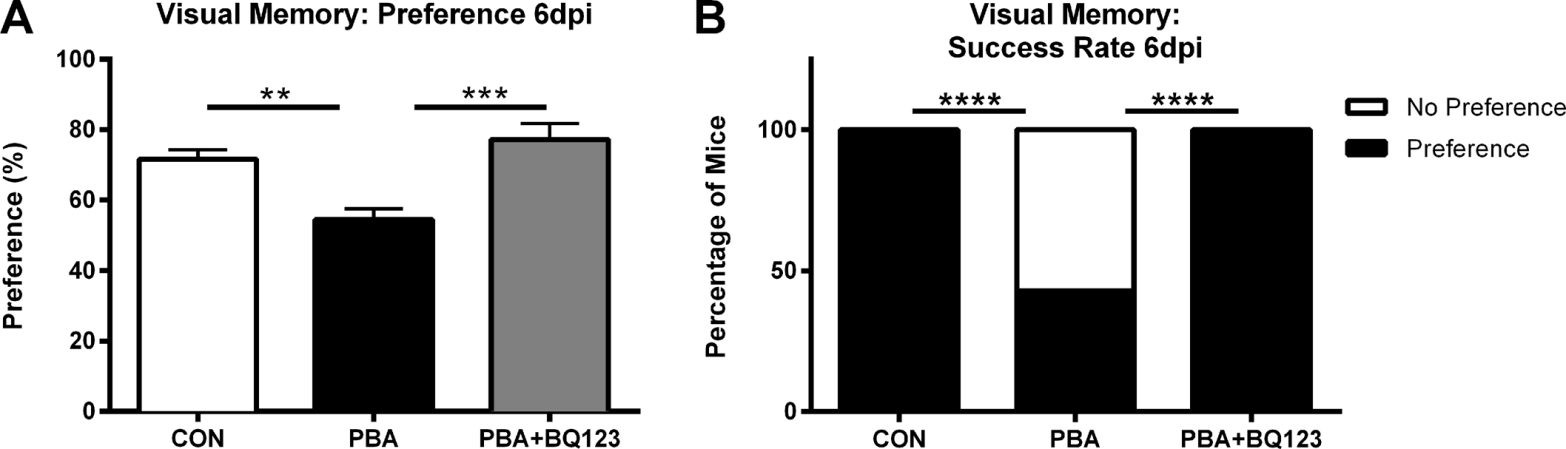
ET-1 contributes to cognitive dysfunction associated with PbA infection. Novel object recognition test of working memory was performed on uninfected and PbA-infected mice at 6dpi. **(A)** Preference scores, expressed as percentage of time spent exploring the novel object, at 6dpi; One-way ANOVA with Tukey’s post-hoc comparisons; ** = p < 0.01, *** = p < 0.001. **(B)** Percentage of mice that preferentially explored the novel object; Chi-square; **** = p < 0.0001. N = 10–13/group.

Our laboratory previously proposed that elevated levels of ET-1 in the brain may contribute to ECM pathogenesis [6, 12]. Corroborating our previous studies, we herein demonstrate that C57BL/6 mice infected with PbA developed visual memory loss during the acute phase of infection (Fig 1) [9]. PbA-infected mice performed significantly worse during the Object Recognition (OR) test than uninfected age-matched controls (Fig 2A; 71.6 ± 3% in uninfected animals vs. 54.4 ± 3% in PbA-infected animals; *P* < 0.01). Furthermore, a significantly lower proportion of infected mice successfully identified the novel object in the OR test when compared to uninfected controls (Fig 2B).

To determine whether blocking the ET_A_ receptor would prevent the ECM-induced neurocognitive impairment, infected mice were treated with the selective ET_A_ receptor blocker (ETARB), BQ123. Infected mice treated with BQ123 performed significantly better in the OR test than infected mice treated with saline (Fig 2A; 77.2 ± 5% vs. 54.4 ± 3%; *P* < 0.001). Unlike untreated infected mice, all PbA-infected mice treated with BQ123 successfully completed the OR test, similar to healthy uninfected controls (Fig 2B).

Memory impairment in ECM mice was not associated with any abnormalities in motor performance or exploratory behavior as all mice had similar motor performance and total exploration time (S2A and S2B Fig).

### ET_A_ receptor antagonism prevents persistent visual memory loss in convalescent mice after successful antimalarial treatment

**Fig 3:**
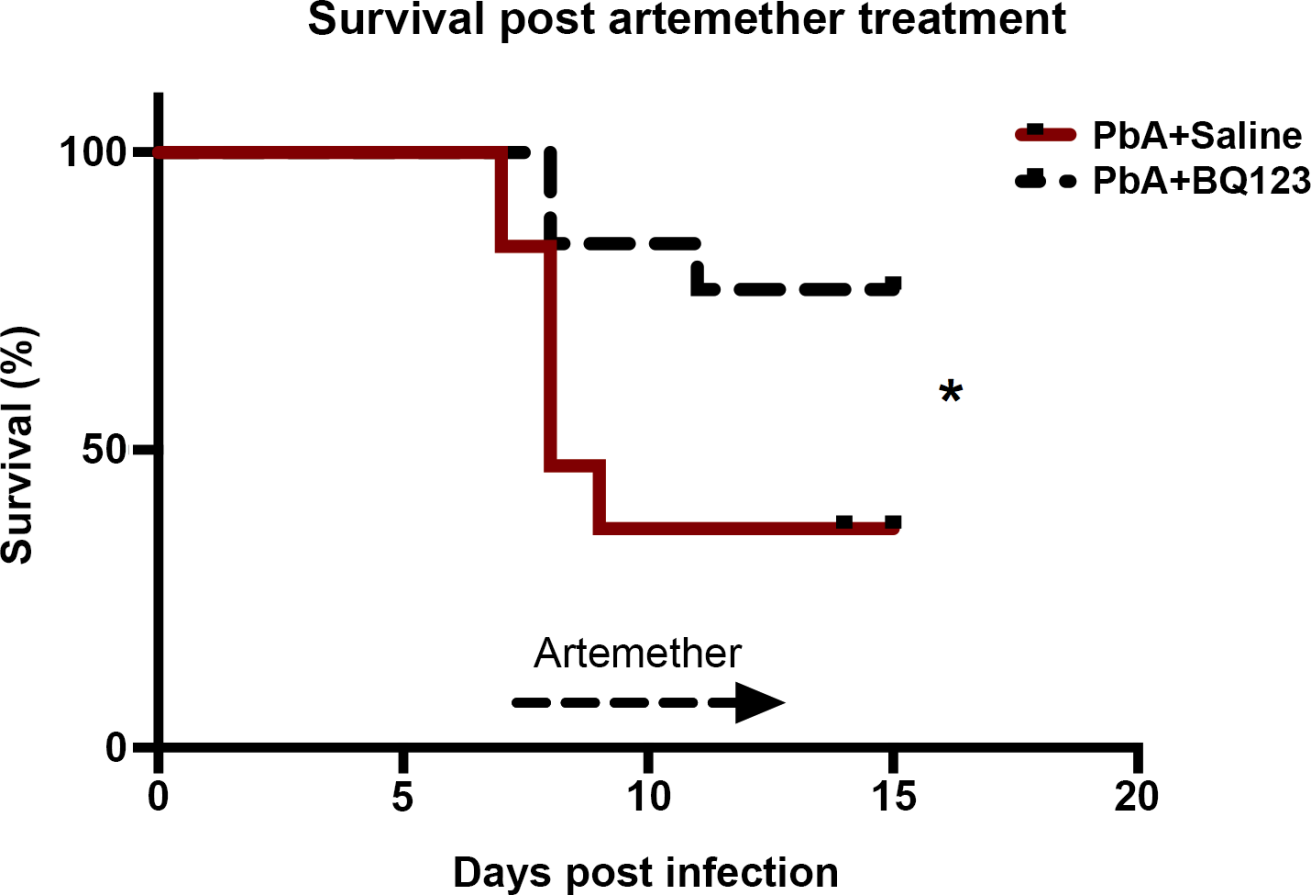
BQ123 effects on survival in convalescent mice after artemether treatment. Despite successful Artemether treatment, mortality remains high in PbA-infected mice. BQ123 adjunctive treatment, in combination with artemether, however, significantly improved survival in PbA mice; Log-rank (Mantel-Cox) test; * = p < 0.05. n = 13–19/group.

**Fig 4:**
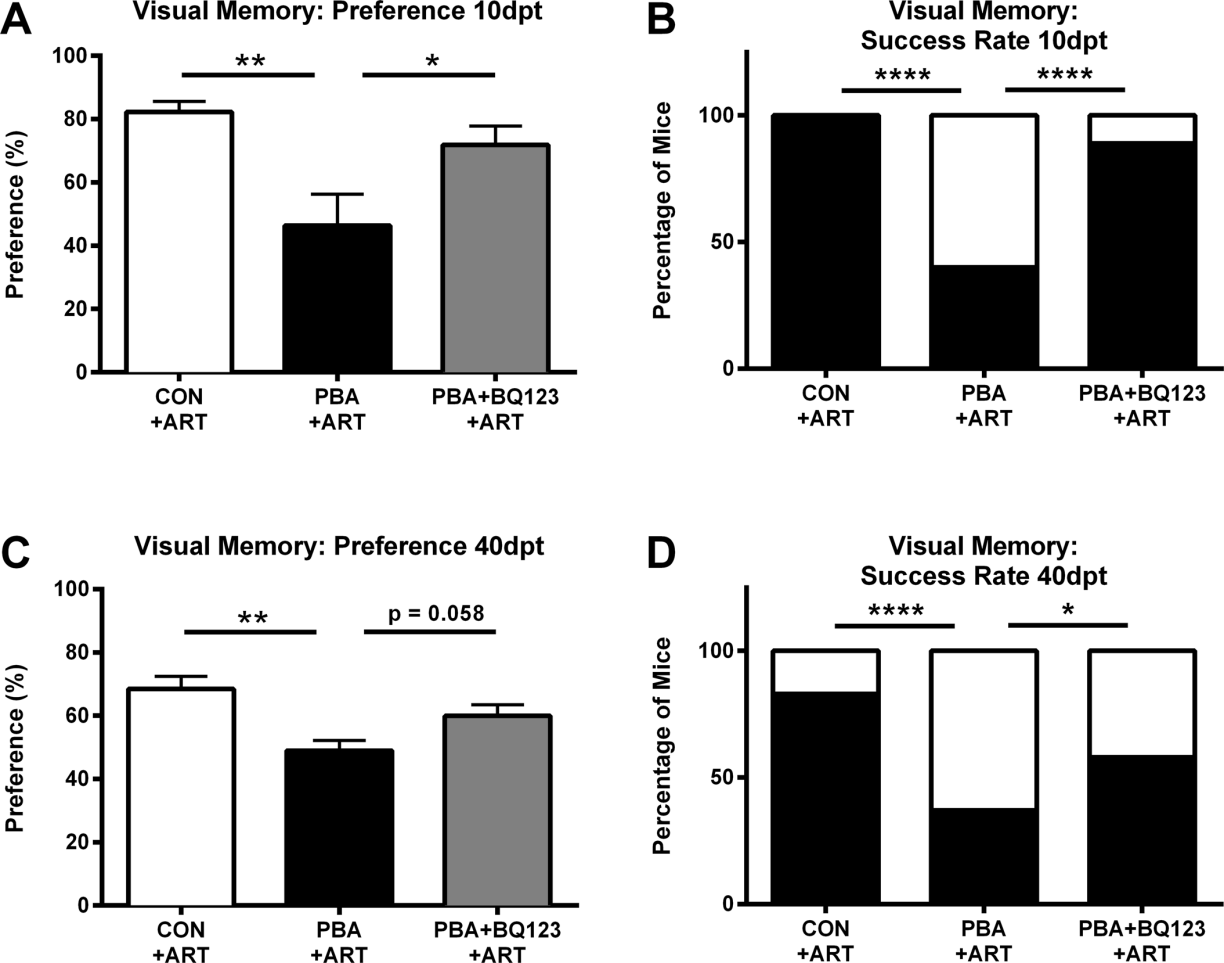
ET-1 contributes to persistent neurological deficits following successful treatment of ECM. Novel object recognition test of working memory was performed on uninfected and PbA-infected saline or BQ123 treated mice 10 and 40 days post artemether treatment (dpt). **(A)** Preference scores at 10 dpt; One-way ANOVA, with Tukey’s analysis; ** = p < 0.01, * = p < 0.05. **(B) 10 dpt.** Percentage of mice that preferentially explored novel object; Chi-square; **** = p < 0.0001. **(C) 40 dpt.** Preference scores at 40 dpt; One-way ANOVA, with Fisher's LSD analysis; ** = p < 0.01, * = p < 0.05. **(D)** Percentage of mice that preferentially explored novel object; Chi-square; **** = p < 0.0001. N = 8–12/group

Both human and experimental CM studies demonstrate that even after successful treatment of malarial infection, cognitive deficits persist [3, 10, 19]. Adjunctive BQ123 treatment in PbA-infected mice treated with the antimalarial agent, artemether, significantly improved survival, aiding in the recovery from ECM (Fig 3). Consistent with our previous publications, artemether-treated PbA-infected mice displayed significant visual memory loss in the OR test of visual memory 10 days after the cessation of anti-malarial treatment, despite parasite eradication (Fig 4A; 46.4 ± 9.9% in PbA infected mice vs. 82.3 ± 3% in uninfected mice; *P* < 0.01). A significantly higher percentage of the artemether-treated PbA-infected mice demonstrated memory impairment than uninfected control mice (Fig 4B). Since treatment with BQ123 prevented cognitive dysfunction during acute illness, we tested whether this protection would be sustained in mice with persistent deficits after antimalarial treatment. Adjunctive therapy with BQ123 prevented visual memory deficits in ECM mice 10 days after antimalarial treatment (Fig 4A and 4B; 71.9 ± 6% in PbA+BQ123+Artemether vs. 46.4 ± 9.9% in PbA+Artemether; *P* < 0.05). BQ123 adjunctive therapy prevented memory impairment in a significantly higher percentage of infected mice treated with artemether (Fig 4B). Mice treated with adjunctive BQ123 exhibited similar preference scores to controls (Fig 4A; 71.9 ± 6% in PbA+BQ123+Artemether vs. 82.3 ± 3% in uninfected mice; *P* = NS).

An estimated 20–25% of patients who survive an episode of CM exhibit long-term cognitive deficits, underscoring an important need for adjunctive therapies in the management of CM [20–23]. As we previously observed, the cognitive deficits detected early after the cessation of anti-malarial therapy were persistent in ECM mice one month later. Forty days after the cessation of artemether therapy, visual memory deficits were evident following a 60-minute retention interval (Fig 4C; 49 ± 3% in infected vs. 68.5 ± 4% in uninfected; *P* < 0.01), and a significantly higher percentage of infected mice exhibited random exploration in the object recognition test (Fig 4D). Preference scores of ECM mice that received adjunctive therapy with BQ123 were comparable to those of uninfected controls (Fig 4C; 68.5 ± 4% in uninfected vs. 59.9 ± 4% in BQ123 treated; *P* = NS). However, BQ123 adjunctive treatment conferred only a partial long-term benefit in convalescent ECM mice. Although a significantly higher percentage of ECM mice treated with BQ123 preferentially explored the novel object than ECM mice treated with artemether alone (Fig 4D; 58% in BQ123-treated vs. 37% in saline treated; *P* < 0.05), BQ123 treatment conferred only a partial protection of visual working memory in artemether treated ECM mice at 40 dpt (Fig 4C; 59.9 ± 4 in BQ123 treated vs. 49 ± 3 in saline treated; *P* = 0.058).

Memory impairment in artemether-treated ECM mice was not associated with abnormalities in motor performance or exploratory behavior as all mice had similar motor performance and total exploration time at 10 and 40 dpt (S3A–S3C Fig).

### ET_A_ receptor antagonism has a positive impact on cerebral microvascular constriction

**Fig 5:**
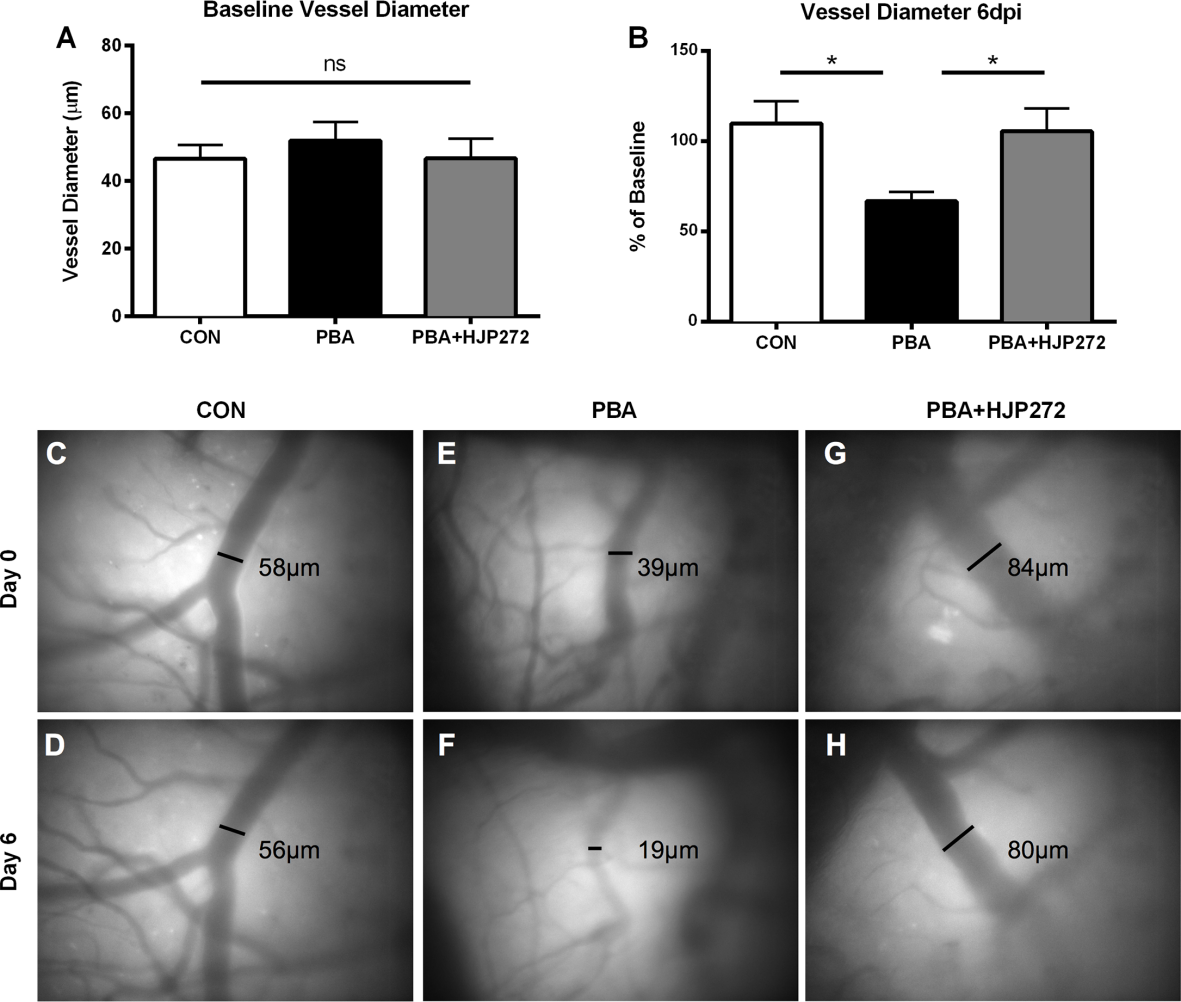
ET-1 contributes to cerebral vascular constriction during ECM. Intravital microscopy was performed to measure vasoconstriction in the cortical microvasculature. **(A)** Baseline vessel diameters; One-way ANOVA with Tukey's analysis. **(B)** Change from baseline in vessel diameters at 6dpi. Vessels are marked at baseline, and the diameter of each marked vessel is measured, then graphed as a percentage its own baseline diameter for each mouse. PbA-infected mice displayed marked decreases in vessel patency relative to uninfected control mice. Administration of HJP272 prevented ECM-induced vasoconstriction; One-way ANOVA with Tukey's post-hoc group comparisons. **(C-H)** Representative images of pial vessels in uninfected **(C,D)**, infected **(E,F)**, and infected HJP272 treated mice **(G,H)** at 0 and 6dpi, respectively. * = p < 0.05. n = 7/group.

Vascular dysfunction including large vessel infarcts, cerebral edema, and impaired tissue perfusion in the retinal microvasculature are important components of CM pathogenesis [24–26]. The neurological impairments present in ECM are often associated with vascular complications including vasoconstriction, vascular collapse, and vascular congestion ultimately leading to disruption of the BBB [7, 27]. In order to determine the association between ET-1 induced microvascular disease and adverse cognitive outcomes during ECM, we assessed cerebral microvascular patency using intravital microscopy through a closed cranial window. As demonstrated in Fig 5A, mean baseline vessel diameters did not vary significantly between the different experimental groups (Fig 5; 46.5 ± 4 in control v. 51.9 ± 5.5 in PbA v. 46.7 ± 5.9 in PbA+HJP272; *P* = NS). In corroboration with previous studies [5, 28], mice with ECM had a significant reduction in pial arterial diameter at 6 dpi, while healthy uninfected animals maintained stable vessel diameters over the course of the experiment (Fig 5B–5F; 66.7 ± 5.1% of baseline in PbA vs. 109.7 ± 12.5% of baseline in control; *P* < 0.05). Treatment with the selective ETARB, HJP-272 prevented the ECM-induced narrowing of the cerebral microvasculature (Fig 5B and 5E–5H; 105.5 ±12.6% of baseline in HJP-272 vs. 66.7 ± 5.1% in PbA; *P* < 0.05).

### ET_A_ receptor antagonism ameliorates endothelial activation and vascular congestion during PbA-infection

**Fig 6:**
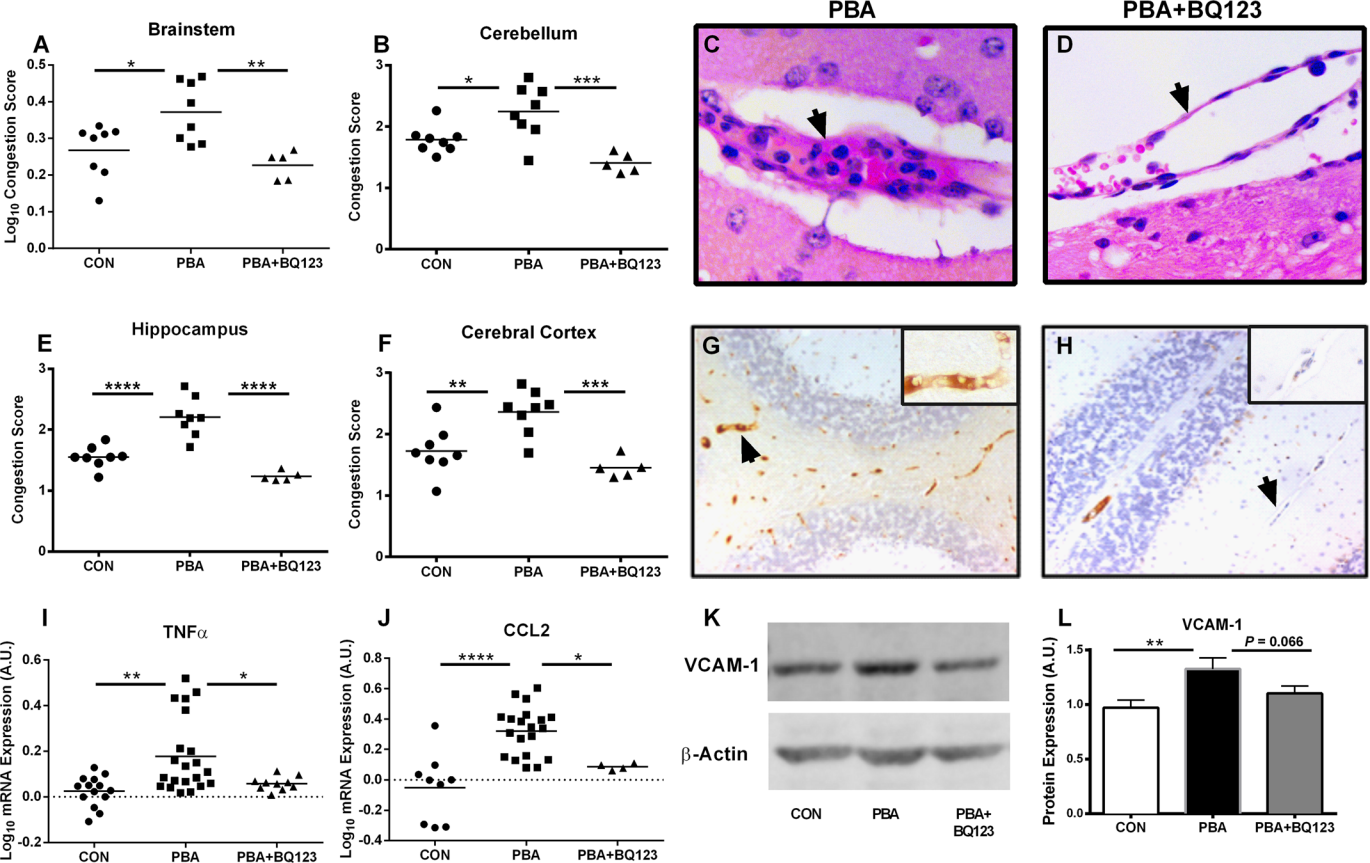
ET-1 mediates cerebral endothelial activation, neuroinflammation and microvascular congestion during ECM. Vascular congestion was assessed by the degree of intravascular obstruction (**A-F**). PbA-infected mice showed a significant increase in cerebral microvascular obstruction compared to uninfected controls in the brainstem (**A**), cerebellum (**B**), hippocampus (**E**), and cortex (**F**). BQ123 treatment significantly reduced the degree of microvascular obstruction of PbA-infected mice in those regions. One-way ANOVA with post-hoc Tukey's comparison. **(C,D; arrows)** Representative histological image of cerebral blood vessel from PbA-infected and PbA-infected BQ123 treated mice. Endothelial activation was determined by VCAM-1 immunostaining (**G,H;** cerebellum shown). **(K)** Representative immunoblot of VCAM-1 protein expression. **(L)** PbA infection resulted in significantly higher VCAM-1 expression in the brain, and this was partially restored to uninfected levels by BQ123. One-way ANOVA with post-hoc Fisher's LSD comparison. (**I,J**) Expression of TNF and CCL2 were quantified by real-time PCR. (**I**) PbA infection resulted significantly higher expression of TNF, and this was prevented by treatment with BQ123. ANOVA with post-hoc Games-Howell comparison. (**J**) Likewise, PbA induced a significant increase in CCL2 expression which was prevented by BQ123. One-way ANOVA with Tukey's comparison. For graphs A, I, J, a logarithmic transformation was performed to yield variance homogeneity and/ or normal distribution. * = p < 0.05, ** = p < 0.01, *** = p < 0.001 and **** p < 0.0001 n = 5–8/group for vascular congestion, TNF and CCL2 analysis; n = 9–15/group for VCAM analysis.

During CM, there is an increase in brain microvascular endothelial cell activation, critical for parasitized red blood cell (pRBC), platelet and leukocyte adhesion, resulting in sequestration, capillary obstruction, localized hypoxia, tissue injury and subsequent neurocognitive impairment or death [29–31]. Endothelial activation and microvascular obstruction are hallmark features of CM, and are associated with coma in patients with CM [24]. Using a semi-quantitative scoring system we assessed the degree of cellular retention within the cerebral vasculature, measured as percent obstruction of vessel lumen. Mice with ECM displayed a significant degree of vessel congestion, measured by the degree of vessel lumen obstruction (Fig 6C and 6D), that was not evident in uninfected mice in several brain regions, including the brainstem (Fig 6A; Log_10_ congestion score: 0.37 ± 0.03 vs. 0.27 ± 0.03; *P* < 0.05), cerebellum (Fig 6B; Score: 2.24 ± 0.15 vs. 1.79 ± 0.08; *P* < 0.05), hippocampus (Fig 6E; Score: 2.2 ± 0.1 vs. 1.55 ± 0.06; *P* < 0.0001), and cerebral cortex (Fig 6F; Score: 2.36 ± 0.13 vs. 1.73 ± 0.14; *P* < 0.01). Treatment with BQ123 prevented cerebral microvascular congestion in the brainstem (Fig 6A; Log_10_ Score: 0.23 ± 0.01 vs. 0.37 ± 0.03; *P* < 0.01), cerebellum (Fig 6B; Score: 1.405 ± .0.7 vs. 2.24 ± 0.15; *P* < 0.001), hippocampus (Fig 6E; Score: 1.24 ± 0.04 vs. 2.2 ± 0.11; *P* < 0.0001), and cerebral cortex (Fig 6F; Score: 1.46 ± 0.08 vs. 2.36 ± 0.13; *P* < 0.001) of PbA-infected mice.

Pro-inflammatory cytokines and cell adhesion molecules have been shown to contribute to the CM disease process by inducing the sequestration of pRBCs and the recruitment and adhesion of leukocytes to vascular beds [32–34]. These inflammatory mediators are increased in response to ET-1 [14, 35, 36], and likely participate in the vascular obstruction observed in our mice. Quantitative real-time PCR at 7 dpi demonstrated that PbA infection resulted in significantly higher expression of tumor necrosis factor-α (TNFα, Fig 6I; Log_10_ mRNA: 0.18 ± 0.04 vs. 0.03 ± 0.02; *P* < 0.01) and the monocyte chemoattractant, CCL2 (Fig 6J; Log_10_ mRNA: 0.32 ± 0.04 vs. -0.05 ± 0.07; *P* < 0.0001) when compared to uninfected mice. In addition, immunoblot and analysis of mice with ECM demonstrate significantly higher vascular cell adhesion protein 1 (VCAM-1) expression in the brain than uninfected controls (Fig 6K and 6L; 1.33 ± 0.1 vs. 0.97 ± 0.07; *P* < 0.01). Immunohistochemical staining of brain sections demonstrated intense VCAM-1 staining in the cerebral vessels of mice with ECM (Fig 6G; arrow, insert). Administration of BQ123 significantly prevented the increased production of TNFα (Fig 6I; Log_10_ mRNA: 0.06 ± 0.01 vs. 0.18 ± 0.04; *P* < 0.05) and in CCL2 (Fig 6J; Log_10_ mRNA: 0.09 ± 0.01 vs. 0.32 ± 0.04; *P* < 0.05) in the brains of PbA-infected mice. However, increases in VCAM-1, induced by PbA infection were only partially reversed by BQ123 treatment (Fig 6L; 1.10 ± 0.07 in PbA+BQ123 vs. 1.33 ± 0.1 in PbA; *P* = 0.066).

### ET_A_ receptor antagonism stabilizes the blood brain barrier during ECM

**Fig 7:**
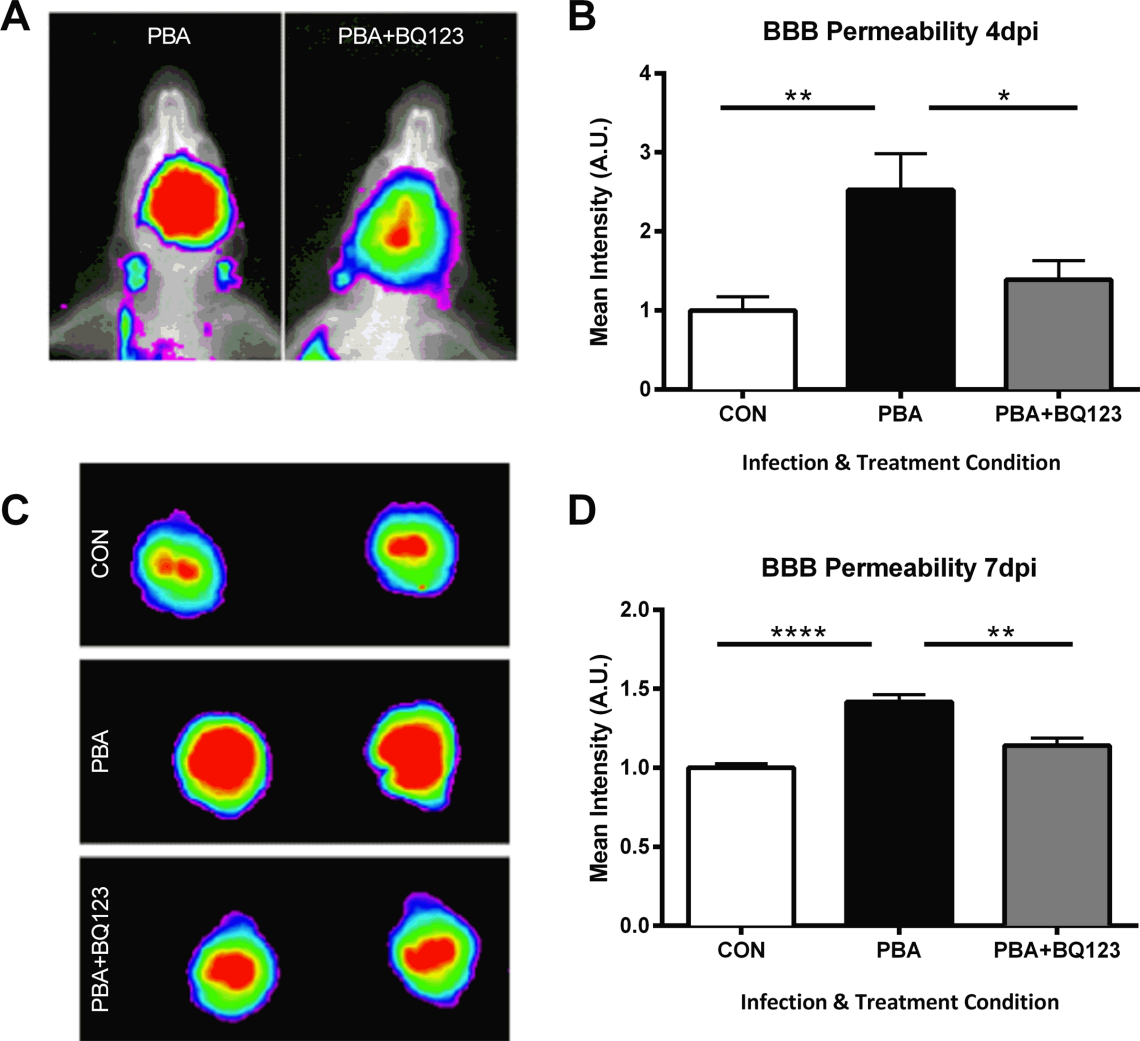
Administration of BQ123 inhibits vascular leakage in PbA-infected mice. Representative *in vivo* (**A**) and *ex vivo* (**C**) brain images of vascular leakage as determined by Tracer-653 fluorescent intensity. PbA-infection enhanced BBB permeability at 4 (**A,B**) and 7dpi (**C,D**). BQ123 prevented BBB damage due to PbA infection at 4 dpi (**B**; p < 0.01) and at day 7 dpi (**D**; p < 0.05). One-way ANOVA with post-hoc Tukey's comparison. n = 6–10/group.

Endothelial activation and vascular obstruction contribute to the loss of vascular integrity during CM pathogenesis. During ECM, ET-1 expression is increased in the brain. ET-1 has been shown to induce disruption of the blood brain barrier (BBB), increasing paracellular permeability [37, 38]. Using a small animal *in vivo* imaging system, we measured BBB leakage by quantifying near-infrared fluorescence intensity of Tracer-653 (Fig 7). PbA infection resulted in impaired vascular integrity, significantly increasing BBB leakage as early as 4 dpi (Fig 7B; 2.52 ± 0.5 in PbA vs. 1 ± 0.2 in control; *P* = < 0.01), and sustained at 7 dpi, during terminal disease (Fig 7D; 1.42 ± 0.05 in PbA vs. 1 ± 0.03 in control; *P* < 0.0001). BQ123 treatment in PbA-infected mice prevented the BBB disruption observed in ECM mice treated with saline (Fig 7), and protected BBB integrity at both earlier (Fig 7B; 1.39 ± 0.2 in PbA+BQ123 vs. 2.52 ± 0.5 in PbA; *P* < 0.05) and later (Fig 7D; 1.14 ± 0.05 vs. 1.42 ± 0.05; *P* < 0.01) stages of disease.

### ET-1 induces cognitive dysfunction in a non-CM model of malarial infection

**Fig 8:**
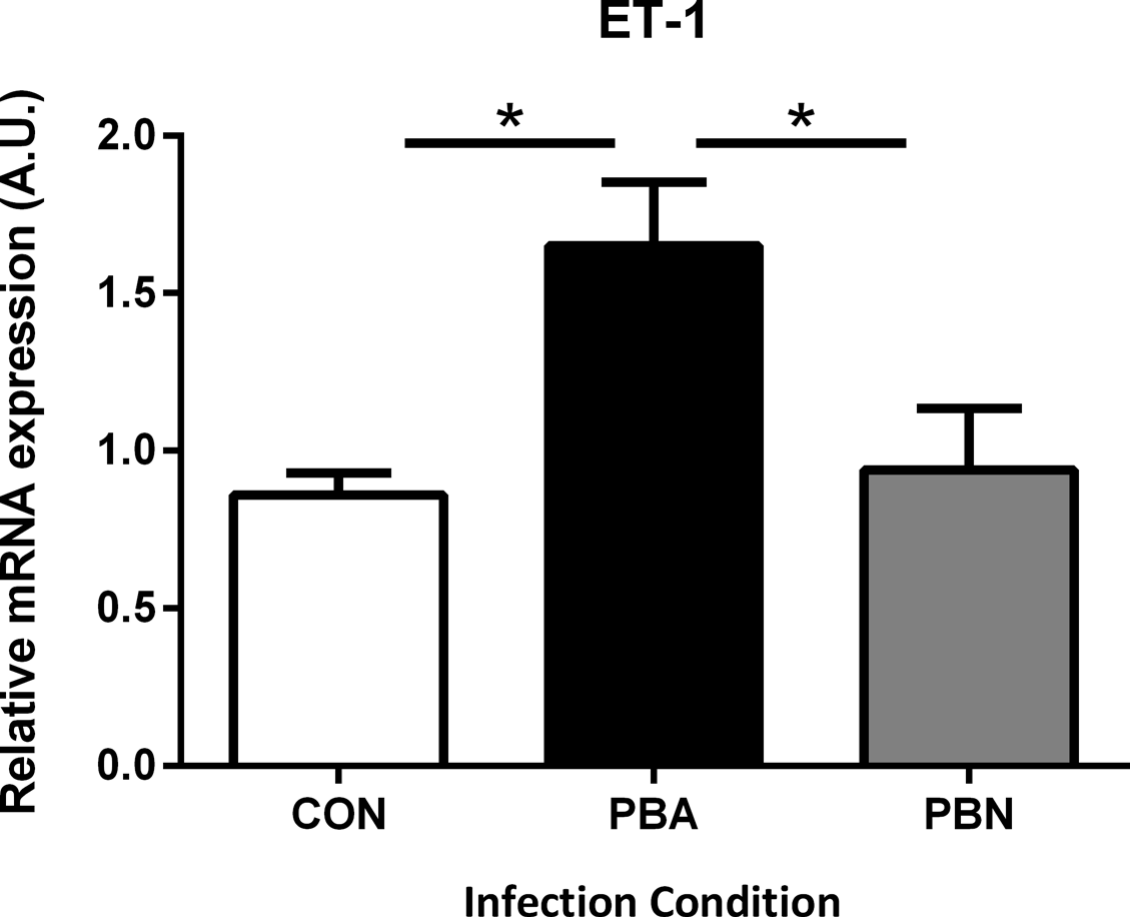
Non-CM does not induce brain ET-1 expression. Quantitative PCR for ET-1 mRNA was performed on whole-brain lysates of uninfected, PbA-infected, and PbN-infected (non-CM) mice. ET-1 was significantly increased in the brains of PbA-infected mice compared to uninfected controls. Levels of ET-1 mRNA were significantly lower in PbN-infected mice when compared to PbA-infected mice (p< 0.05). ET-1 mRNA levels in PbN brain were no different from control. One-way ANOVA. with post-hoc Tukey's comparison. n = 5–7/group.

Infection of C57BL/6 mice with *P*. *berghei* NK65 (PbN), a model often used as a non-CM control for PbA induced ECM, results in a syndrome of severe malaria, but does not cause ECM [8, 39]. While PbA-infected mice display significantly higher expression of all the components of the ET-1 pathway when compared to uninfected controls [6], PbN-infected mice had significantly lower ET-1 expression than ECM mice (Fig 8; 0.94 ± 0.19 PbN vs. 1.65 ± 0.2 PbA; *P* < 0.05), with levels comparable to those in uninfected controls (Fig 8; 0.94 ± 0.19 vs. 0.86 ± 0.07; *P* = NS).

**Fig 9:**
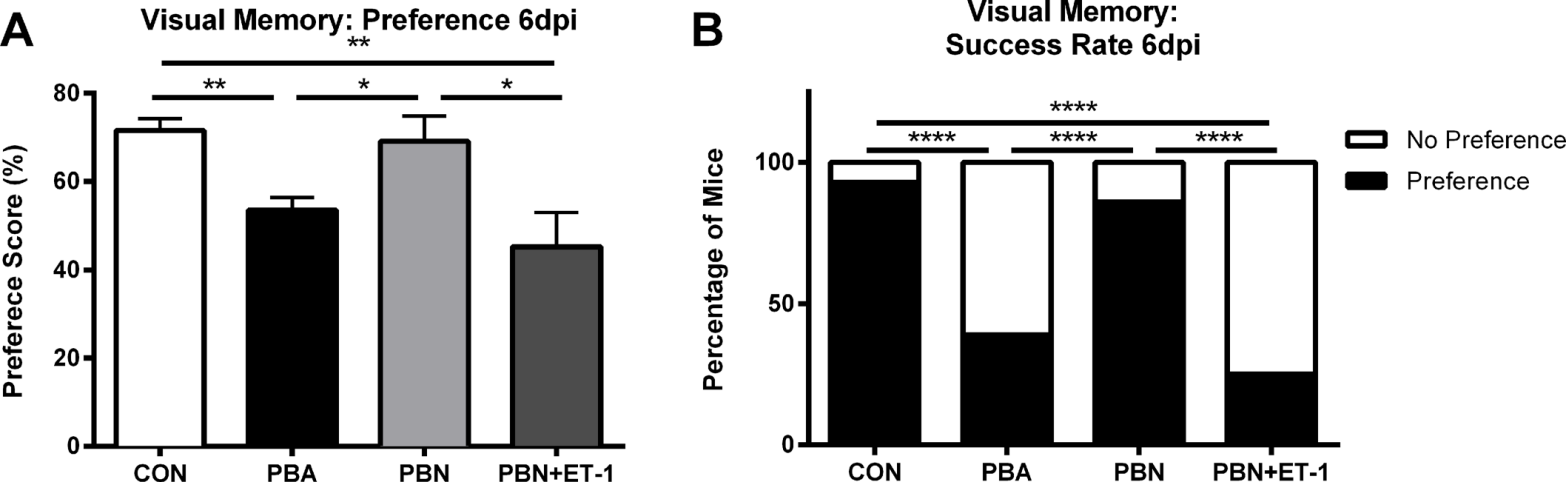
ET-1 induces cognitive dysfunction in non-CM mice. Visual memory testing was performed on PbN-infected (non-CM) and PbN-infected ET-1 treated mice. Non-CM mice do not display visual memory impairments (**A,B**). **(B)** A significantly higher proportion of PbN mice passed the object recognition test when compared to PbA mice (p < 0.0001). **(A)** Exogenous ET-1 treatment induced cognitive dysfunction in PbN-infected mice (p < 0.05). **(B)** In addition, PbN ET-1 treated mice performed significantly worse than PbN saline treated mice (p < 0.0001). One-way ANOVA was performed for preference scores in **A**, and Chi-square was performed for success rate in **B**. n = 4–20/group.

Unlike ECM mice, PbN-infected mice did not exhibit visual memory deficits at 6dpi (Fig 9A; 53.53 ± 2.9% in ECM vs. 69.07 ± 5.8% in non-CM, PbN-infected mice; *P <* 0.05). In addition, a greater proportion of non-CM mice were able to successfully completed the object recognition test of visual memory compared to ECM mice (Fig 9B; 86% non-CM vs. 39% ECM; *P* < 0.001).PbN-infected mice performed as well as uninfected controls in the object recognition test (Fig 9A; 69.07 ± 5.8% in PbN mice vs. 71.59 ± 2.7% in uninfected controls; *P* = NS). To establish whether the deleterious effects of ECM were in fact induced by increased production of ET-1, we treated PbN-infected mice with exogenous ET-1 and tested their visual memory. Similar to PbA-infected mice, ET-1 treated non-CM mice displayed impaired visual learning (Fig 9A; 45.22 ± 7.7% PbN+ET-1 vs. 71.59 ± 2.7% controls; *P* < 0.01). A significantly lower proportion of non-CM mice treated with ET-1 were able to successfully complete this memory task when compared to PbN-infected mice treated with saline (Fig 9B; 86% in PbN infected mice vs. 25% in PbN+ET-1 mice; *P* < 0.001), supporting our theory that cognitive impairment in ECM is induced by ET-1.

## Discussion

CM has long been thought to cause a vascular inflammatory process in the brain [11, 27]. Yet a comprehensive delineation of the precise mechanisms by which malarial infection promotes cerebral vasculopathy, BBB disruption, CNS pathology, and the ensuing neurocognitive deficits remains elusive; impeding successful efforts to develop adjunctive therapies. Previous research has shown that ECM results in imbalances in vascular tone and vasoactive mediators [27, 40, 41]. Furthermore, pRBCs, platelets and leukocytes have been shown to sequester to cerebrovascular beds secreting cytokines and chemokines further obstructing cerebral blood flow triggering a vasoactive, pro-inflammatory environment [24, 31, 40, 42–44]. This environment leads to neurological and cognitive complications, which are known to persist long after the resolution of infection [3, 4, 21, 22].

Increased concentrations of ET-1 have been reported in patients with *P*. *falciparum* infection [17, 45]. Interestingly, TNF-α reduces cerebral blood volume, and disrupts the BBB and tissue homeostasis in Wistar rats in an endothelin dependent manner [13]. Our group previously demonstrated that ET-1 was elevated in the brains of mice with ECM [6]. In the current study, we demonstrated that cerebrovascular constriction, the activation of certain cytokines and chemokines, vascular congestion, and BBB disruption, as well as the ensuing increase in mortality and acute and chronic visual memory impairments during ECM were mitigated by treatment with an ET_A_ receptor blocker. More importantly, our observations were substantiated by the induction of visual memory deficits in non-CM (PbN-infected) mice upon administration of exogenous ET-1. While the direct effects of *P*. *falciparum* infection on the expression and function of ET receptors have not previously been investigated; altogether, these data strongly support a role for ET-1 in the pathogenesis of ECM. Treatment with ET_A_ receptor antagonists have been effective in preventing cognitive impairment in other animal models [46, 47]. Although we acknowledge that in the absence of genetic knockout experiments to confirm selectivity, the possibility of off-target effects of ET-1 receptor antagonism are inherent limitations in the study, the protective effects of ET_A_ receptor antagonism with ECM observed herein can be ascribed to a reduction in malaria-induced cerebrovascular constriction and congestion as well as prevention of ET-mediated endothelial activation and BBB leakage.

A major physiological role of ET-1 is the regulation of vascular tone. ET-1 binding to the ET_A_ receptor induces vasoconstriction and reduction of CBF by inhibiting nitric oxide (NO) production and increasing intracellular concentrations of Ca^2+^ in vascular smooth muscle cells [28, 48–50]. Previously, our laboratory group demonstrated an association between increased ET-1 expression and a decrease in CBF in mice with ECM [6]. Previous intravital microscopic studies demonstrate that ECM resulted in the constriction of pial vessels in the brain, contributing to diminished CBF and vascular collapse reminiscent of subarachnoid hemorrhage [7, 41]. Using intravital microscopy through a closed cranial window we demonstrated that ET_A_ receptor antagonism prevented vasoconstriction during ECM. ET-1 has been shown to increase the concentration of intracellular Ca^2+^ from internal and external stores, mediating cell death [51]. In several studies, blockage of the ET_A_ receptor prevents intracellular mobilization of Ca^2+^, and reduces intracellular accumulation of Ca^2+^, ultimately preventing vessel constriction [52, 53]. Vasoconstriction in CM is likely a result of ET-1 mediated increases in Ca^2+^ mobilization in the CNS [7, 27, 41].

In the current studies, ET_A_ antagonism also resulted in suppression of the infection-induced inflammatory response. Furthermore, ET_A_ antagonism blunted the associated vascular obstruction resulting from leukocyte, pRBC and platelet accumulation. VCAM-1 expression was only partially reduced in the brains of PbA infected mice treated with the ETARB BQ123 toward uninfected levels. This may indicate that additional factors beyond ET-1 are involved in the induction of VCAM-1 during ECM, or alternatively, that increases in VCAM-1 could have limited reversibility during PbA infection. Activation of the cerebrovascular endothelium contributes to the development of both human and murine models of CM [30]. In this regard, cerebral vascular congestion has been shown to correlate with coma in the setting of CM [24]. The upregulation of cytokines, such as TNFα, which results in the increased expression of leukocyte binding adhesion molecules and chemokines [54], is a hallmark of CNS infection [34]. In this regard, Wassmer et. al. demonstrated that cultured vascular endothelial cells isolated from patients with CM significantly upregulated their production of CCL-2 in response to TNFα stimulation [55]. This increased production may be dependent on ET-1. The ET-1-mediated increase in CCL-2 production observed in our model likely increases the recruitment and diapedesis of immune cells into the brain parenchyma, further exacerbating neuroinflammation and ultimately disrupting BBB integrity. Administration of ET_A_ receptor antagonists in our mice prevented ECM-induced accumulation of cells in the brain microvasculature, ultimately maintaining cerebral vascular patency.

Our observations support the concept that ET-1 plays a central pathogenic role in the development of CM. The current study provides evidence that endothelial activation and inflammation, as well as vascular constriction, occlusion, and permeability contribute to the cognitive impairment and mortality following PbA-infection. We propose that ET_A_ antagonism may be beneficial in CM by inhibiting the vasoconstriction, endothelial activation, and BBB disruption that occur with infection. The efficacy of this therapy in our study is likely due to the observed protective effects on the microvascular endothelium, as the induction of cellular adhesion molecules has been associated with ETA signaling on endothelial cells [14, 56], and on the inflammatory response in the brain [13, 35, 36, 55]. By reversing endothelial activation and neuroinflammation, ETARB may restore vascular patency and CBF, ultimately improving oxygen delivery to the brain. We previously observed that with resolution of acute *P*. *berghei* infection, there remained significant brain microvascular damage during the early convalescent period [12], which was resolved by 40 dpt [10]. As BQ123 does not cross the BBB [57], the partial protection of neurological function at 40 dpt, conferred by BQ123 adjunctive therapy in the current study, may be due to possible residual CNS damage downstream of the microvasculature. Additional studies, directly targeting CNS ET-1 and ET receptors, should help determine the full extent of ET-1 induced CNS damage during ECM.

Since the discovery of ET-1 in the late 1980s it has been implicated in the pathogenesis of many disease states. These include congestive heart failure, subarachnoid hemorrhage, pre-eclampsia, cancer, hypertensive crisis and primary pulmonary hypertension [58]. In the realm of infectious diseases bedsides malaria ET -1 has also been linked to bacterial sepsis, meningitis and pneumonia [28]. In clinical trials, ET receptor antagonism has been promising in the management of primary hypertension [58], skin microcirculation and limb ischemia in diabetic microangiopathy [59, 60], and ET receptor blockers have been the mainstay of therapy in the management of primary pulmonary hypertension [58, 61].

While we recognize that further studies are required to determine other mechanisms by which ET-1 participates in persistent cognitive deficits or in mortality during acute illness, we conclude that there is therapeutic potential in using the ET system as a target for adjunctive therapy. In conjunction with anti-malarials, pharmacologic antagonism of the ET system may be of value in the management of CM.

## Materials and Methods

### Ethics statement

All experimental protocols were carried out in strict accordance with the recommendations in the Guide for the Care and Use of Laboratory Animals of the National Institutes of Health, reviewed and approved by the Institutional Animal Care and Use Committee of the Albert Einstein College of Medicine (protocol number: 20130602). All efforts were made to minimize suffering.

### Mice, parasites, RMCBS and drug treatment

A timeline of animal infection, drug treatment schedule, and behavior testing is delineated in S4 Fig. Six to 8 week old C57BL/6 mice (Jackson Laboratories, Bar Harbor, ME) were intraperitoneally (i.p.) infected with 10^6^ *P*. *berghei* ANKA (PbA)- or *P*. *berghei* NK65 (PbN)- parasitized RBCs (pRBCs) to either induce ECM or severe malaria without ECM (non-CM) respectively [10, 62]. Mice injected with uninfected RBCs served as a healthy controls.

Disease severity was scored according to a grading system described by *Carroll et al* [63]. A quantitative rapid murine coma and behavior scale (RMCBS) was used to assess the development of ECM. The RMCBS comprised of 10 parameters in which hygiene-related behavior, gait, body position, exploratory behavior, and balance [63] were monitored daily throughout the study for health status, as were weight and temperature. Parasitemia (percentage of pRBCs), assessed by examination of tail blood smears stained with Giemsa, was also monitored daily.

Intraperitoneal osmotic minipumps (model #1002, Alzet) were placed into PbA-infected mice, which were then randomly assigned to receive continuous infusion of either BQ123 (120/ug/kg/d, selected based on dose titration experiments; Sigma) or vehicle (sterile normal saline) via the pumps for a total of 10 days. Mice undergoing cranial window surgery were treated once daily with either 50 mg/kg of HJP-272 via IP injection or saline, initiated at 3dpi for a total of 10 days. In some experiments, PbA-infected mice were allowed to manifest the signs of ECM, as previously described [10], then treated with Artemether (at a dose of 50mg/kg; Kind gift from Dafra Pharma GmbH, Switzerland) dissolved in coconut oil and administered by i.p. injection, for a total of 5 days [12].

In a separate set of experiments PbN-infected mice were treated daily with either exogenous ET-1, dissolved in normal saline (7.5ug/mouse; Millipore), or with normal saline, administered by i.p. injection beginning at day 3 after infection.

Mice were sacrificed and intracardially perfused with ice-cold PBS. Brains were excised and stored at -80°C for analysis.

### Cognitive tests

Behavioral tests were performed to determine early and persistent cognitive deficits present during ECM [9, 10]. Animals underwent cognitive testing at 3 different time points: 6 days post infection (dpi), 10 days and 40 days after the cessation of Artemether treatment (S4 Fig). Object recognition tests were performed to assess visual memory.

During the object recognition test animals underwent a training and test trial, as previously described [9, 10, 64]. This test is based off the inherent tendency of mice to preferentially explore novel objects [9, 10, 64]. In the training trial mice were gently placed in an open field with two identical objects. Mice were allowed to habituate and object exploration of each object was recorded. Mice were then returned to their home cage for a retention interval of 60min. After a 60min retention interval mice were returned to the open field with the one familiar object and one novel object. Again exploration of each object was recorded.

Healthy mice preferentially explore novel objects. Data are represented as the amount of novel object exploration divided by the total time exploration of both objects. A preference score of 50% indicates chance performance, whereas a score greater than 55% indicates novel object preference. Test objects were counterbalanced to prevent confounds of preferential exploration of an object. Examiners were blind to infection and treatment conditions of the mice.

### Cranial window surgery and intravital microscopy

A closed cranial window was placed as previously described [7]. Briefly, mice were anesthetized, the scalp was then removed and a craniotomy of 3–4 mm in diameter was created, using a surgical drill. The exposed tissue was covered with a 5mm glass coverslip. Two weeks after surgery, a panoramic photograph of vessels was taken and a map of selected vessels was created. Approximately 7–10 vessels were imaged in each animal at baseline and 6 dpi. Vessel diameters at 6 dpi were compared to baseline measurements in the same mice for comparison. Data are represented as percent change in vessel diameter to baseline measurements.

### Blood brain barrier disruption

Blood brain barrier (BBB) disruption was assessed as previously described [65]. Briefly, experimental mice received a tail vein injection of Tracer-653 (Molecular Targeting Technologies, Inc.) diluted in PBS [65]. Four hours later mice were anesthetized with ketamine and xylazine and placed inside an *in vivo* imaging system (IVIS) Kodak Image Station 4000MM PRO (Carestream Health) equipped with a CDD camera. In some experiments, mice were anesthetized, intracardially perfused and *ex vivo* imaging was performed on excised brains. Acquired images were analyzed with Carestream MI 5.3.17476 Application Software.

### Preparation of RNA samples and semiquantitative real-time PCR

As described elsewhere, total RNA was extracted from the cerebrum and reverse-transcribed to cDNA [6]. CCL2, TNFα and ET-1 transcription levels were assessed by real-time RT-PCR using Sybr Premix Ex Taq (TaKaRa). The following primers were used: 5'- GAGTAGGCTGGAGAGCTA- AAGAG -3' (forward) and 5'- AGGTAGTGGATGCATTAGTTCAG -3' (reverse) for CCL2; 5’- GAGAAAGTC AACCTCCTCTCTG -3’ (forward) and 5’- GAAGACT-CCTCC- CAGGTATATG -3’ (reverse) for TNFα; 5’ CTGCCACCTGGACATCATC 3’(forward) and 5’ TCCTTCCTTCCACCAGCTG 3’ (reverse) for ET-1 and 5’- AACTTTGGCATTGTG-GAAGG -3’ (forward) and 5’- ACACATTGGGGGTAGGAACA -3’ (reverse) for GAPDH. mRNA values were normalized to those of GAPDH.

### Immunoblotting

Western blot of whole brain homogenates was performed as described earlier [62]. Protein levels of vascular cell adhesion molecule- 1(VCAM-1; Abcam, Cambridge, MA), and β-actin (Abcam) were measured in experimental mice.

### Histology and immunohistochemistry

Mice were anesthetized by isoflurane inhalation and perfused through the heart with saline. Brains were excised, fixed and embedded in paraffin. Brains were cut into 4 μm sagittal sections and stained with hematoxylin-eosin. A congestion score adapted from Fauconnier et. al. was performed to quantify the degree of obstruction in the cerebral vasculature after perfusion [66]. Briefly, approximately 500 vessels were analyzed per group. Congestion scores were based on a scale of 0–4; 0 = No cells present within the vessel lumen; 1 = vessels with a minimal amount of cells, including RBCs, platelets, and adherent leukocytes, obstructing less than 10% of the vessel lumen; 2 = vessels with a moderate degree of cellular obstruction, with 10% to 50% occlusion of the vessel lumen; 3 = vessels with an extensive amount of cellular obstruction, with 50% to 90% occlusion of the vessel lumen; 4 = completely occluded vessel lumen. Immunohistochemistry was performed on sagittal sections as previously described [6]. VCAM-1 (Santa Cruz, Dallas, TX) expression was assessed in experimental animals. Isotype control staining was performed to account for non-specific binding to tissue.

### Statistical analysis

Statistical analyses were performed using one-way ANOVA, with post-hoc group comparisons tests. In cases where the variances between samples were significantly different, and/ or the sample distributions were significantly skewed, i.e., violated normal Gaussian curves, a logarithmic transformation was performed on the samples to yield variance homogeneity and/ or normal distribution, and ANOVA were calculated on the transformed data. Success rates in cognitive tests were analyzed by Chi Square tests. Results are shown as mean ± S.E.M. Analyses were conducted with the GraphPad Prism Software (GraphPad Software Inc., La Jolla, CA) or with the IBM SPSS Statistics v23 software (IBM Corporation, Armonk, NY). A *P* < 0.05 was considered significant.

Additional methods can be found in S1 Text.

## Supporting Information

S1 Fig BQ123 effects on body weight and temperature Changes in body weight **(A)** and temperature **(B)** were assessed in uninfected and PbA-infected mice treated with saline or BQ123. * = p < 0.05 and **** = p < 0.005 by two-way ANOVA. * = Con vs. PbA; # = Con vs. PbA+BQ123. n = 10/group.
(TIFF)

S2 Fig Motor coordination, exploratory behavior and total exploration time in mice 6 dpi. (**A**) Using measurements from the rapid murine coma and behavior scale, gait, balance, corners of the cage explored within 90 seconds, and motor performance, were analyzed. Data were graphed by RMCBS score. (**B**) Total exploration time in the open field was quantified. One-way ANOVA. n = 10/group.
(TIF)

S3 Fig Motor coordination, exploratory behavior and total exploration time in mice 10 and 40 dpt. (**A**) Using measurements from the rapid murine coma and behavior scale, gait, balance, corners of the cage explored within 90 seconds, and motor performance, were analyzed at 10 dpt. Data were graphed by RMCBS score. (**B**) Total exploration time in the open field was quantified at 10 dpt and at 40 dpt. One-way ANOVA. n = 8–12/group.
(TIF)

S4 Fig Timeline of animal infection, drug treatment schedule, and behavior testing. At day 0, mice were injected with either parasitized RBCs (pRBCs) or uninfected RBCs. Treatment with BQ123 was started at 3 dpi for a total of 10 days. For acute illness assessment, cognitive testing was performed 6 dpi (acute cognitive testing). Treatment with artemether was initiated after day 7 and continued for 5 days. Cognitive testing was again performed 10 days after the cessation of treatment (cognitive testing 10 dpt), and again 40 days after the cessation of treatment (cognitive testing 40 dpt) to examine long-term cognitive function.
(TIF)

S1 Text Additional methods. (DOCX)
