# Supplementary figures and images for: Endothelin-1 Mediates Brain Microvascular Dysfunction Leading to Long-Term Cognitive Impairment in a Model of Experimental Cerebral Malaria
Source: PLoS Pathog. 2016 Mar 31;12(3):e1005477. doi: 10.1371/journal.ppat.1005477 (PMC4816336; doi:10.1371/journal.ppat.1005477)

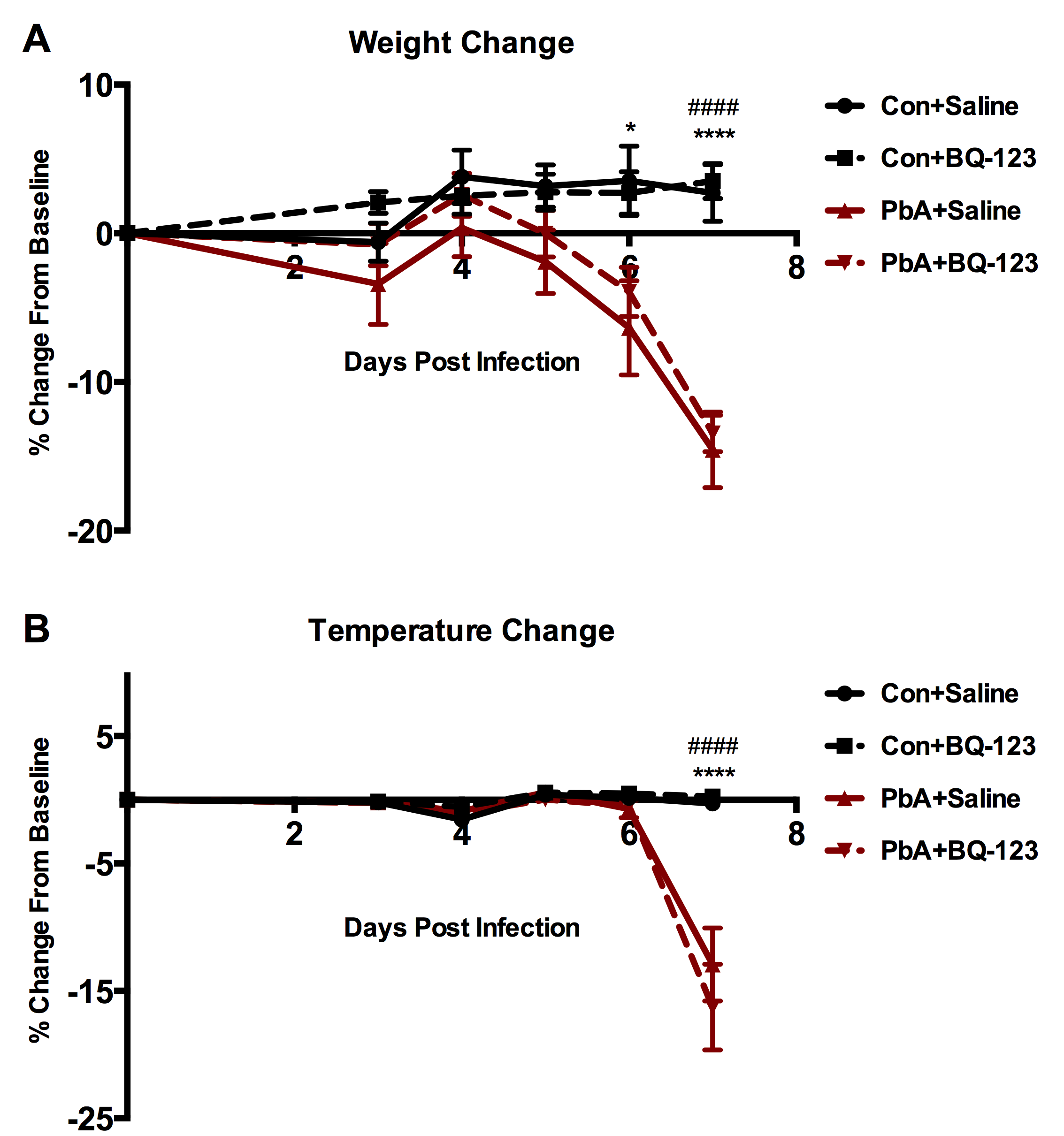

Supplement: S1 Fig — Changes in body weight (A) and temperature (B) were assessed in uninfected and PbA-infected mice treated with saline or BQ123. * = p < 0.05 and **** = p < 0.005 by two-way ANOVA. * = Con vs. PbA; # = Con vs. PbA+BQ123. n = 10/group. (TIFF) [file ppat.1005477.s001.tiff]

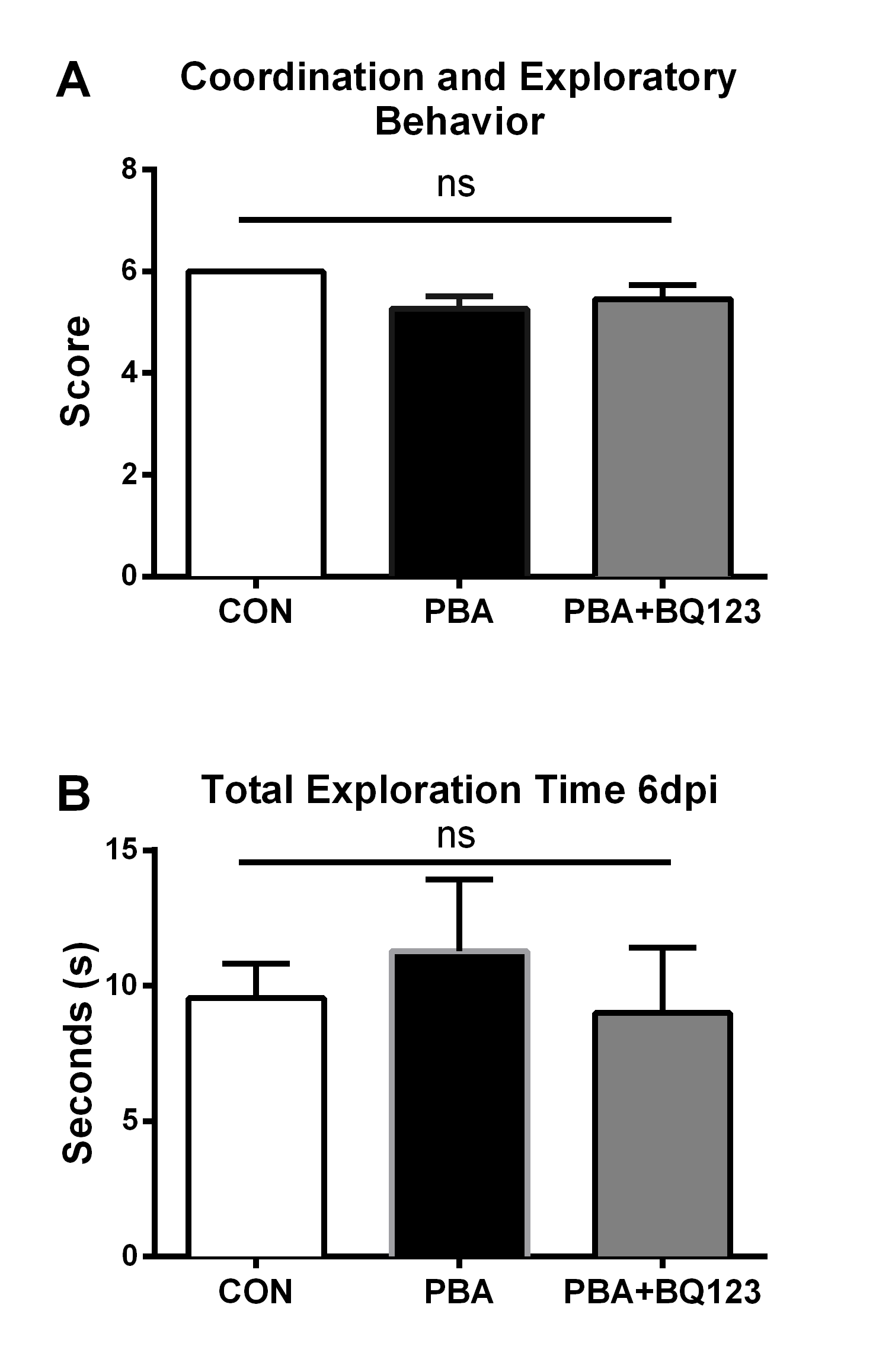

Supplement: S2 Fig — (A) Using measurements from the rapid murine coma and behavior scale, gait, balance, corners of the cage explored within 90 seconds, and motor performance, were analyzed. Data were graphed by RMCBS score. (B) Total exploration time in the open field was quantified. One-way ANOVA. n = 10/group. (TIF) [file ppat.1005477.s002.tif]

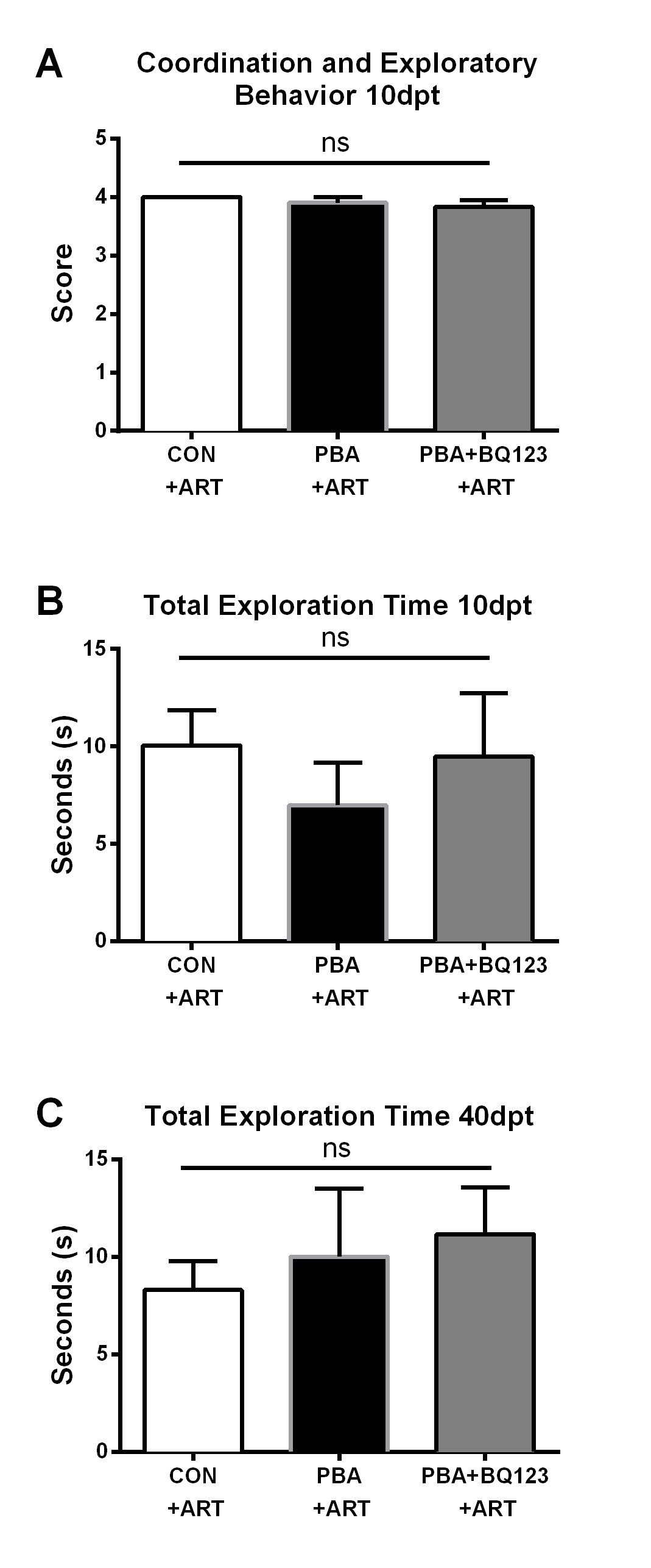

Supplement: S3 Fig — (A) Using measurements from the rapid murine coma and behavior scale, gait, balance, corners of the cage explored within 90 seconds, and motor performance, were analyzed at 10 dpt. Data were graphed by RMCBS score. (B) Total exploration time in the open field was quantified at 10 dpt and at 40 dpt. One-way ANOVA. n = 8–12/group. (TIF) [file ppat.1005477.s003.tif]

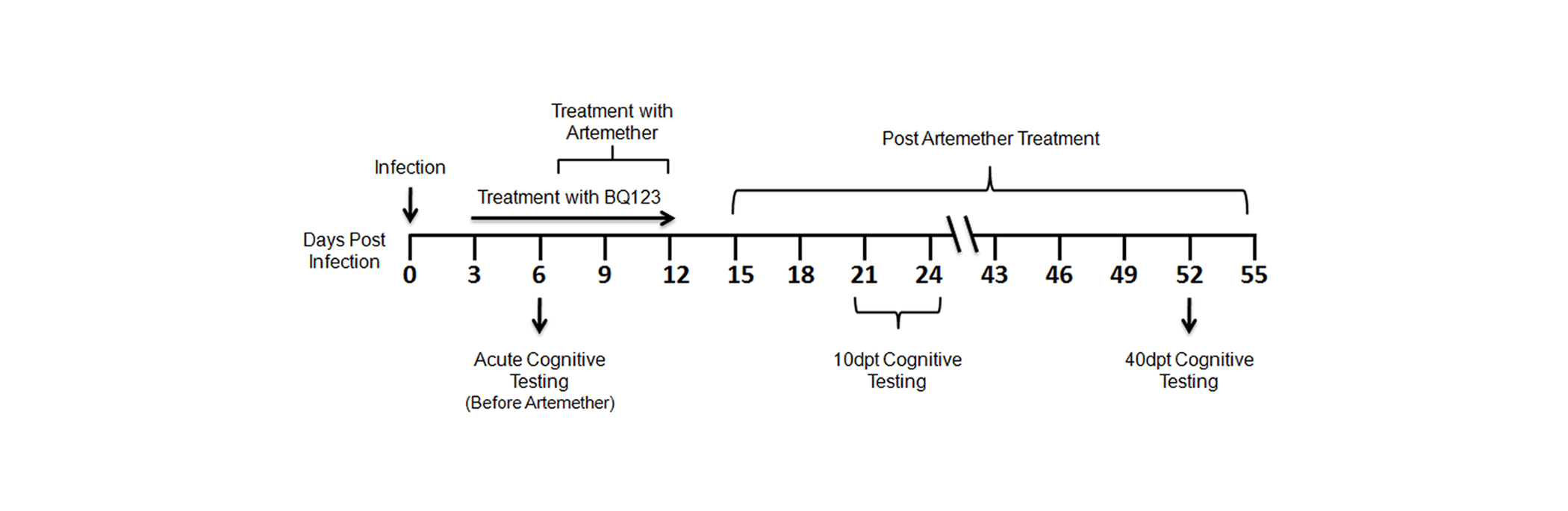

Supplement: S4 Fig — At day 0, mice were injected with either parasitized RBCs (pRBCs) or uninfected RBCs. Treatment with BQ123 was started at 3 dpi for a total of 10 days. For acute illness assessment, cognitive testing was performed 6 dpi (acute cognitive testing). Treatment with artemether was initiated after day 7 and continued for 5 days. Cognitive testing was again performed 10 days after the cessation of treatment (cognitive testing 10 dpt), and again 40 days after the cessation of treatment (cognitive testing 40 dpt) to examine long-term cognitive function. (TIF) [file ppat.1005477.s004.tif]
