# Supplementary material for: Endothelin-1 Mediates Brain Microvascular Dysfunction Leading to Long-Term Cognitive Impairment in a Model of Experimental Cerebral Malaria
Source: PLoS Pathog. 2016 Mar 31;12(3):e1005477. doi: 10.1371/journal.ppat.1005477 (PMC4816336; doi:10.1371/journal.ppat.1005477)
Supplement: S1 Text — (DOCX) [file ppat.1005477.s005.docx]

**SUPPORTING INFORMATION**

**METHODS**

**Mice, Parasites, RMCBS and Drug Treatment.** All experiments used methods and guidelines approved by the Institutional Animal Care and Use Committee of Albert Einstein College of Medicine. Six to 8 week old C57BL/6 mice (Jackson Laboratories, Bar Harbor, ME) were intraperitoneally (i.p.) infected with 10^6^ *P. berghei* ANKA (PbA)- or *P. berghei* NK65 (PbN)- parasitized RBCs (pRBCs) to either induce ECM or severe malaria without ECM (non-CM) respectively [[10](#_ENREF_10), [62](#_ENREF_62)]. Mice injected with uninfected RBCs served as a control.

Disease severity was scored according to a grading system described by *Carroll et al*. A quantitative rapid murine coma and behavior scale (RMCBS) was used to assess the manifestation of ECM. The RMCBS comprised of 10 parameters in which hygiene-related behavior, gait, body position, exploratory behavior, and balance were assessed [[63](#_ENREF_63)]. Using measurements from the rapid murine coma and behavior scale, motor coordination and exploratory behavior, which included assessment of gait, balance, corners of the cage explored within 90 seconds, and motor performance, were analyzed. Data were graphed by RMCBS score.

Intraperitoneal osmotic minipumps (model #1002, Alzet) were placed into PbA-infected mice, which were then randomly assigned to receive continuous infusion of either BQ123 (120/ug/kg/d, selected based on dose titration experiments; Sigma) or vehicle (sterile saline) via the pumps for a total of 10 days. Mice undergoing cranial window surgery for the intravital microscopic evaluations were treated once daily with either 50 mg/kg of HJP272 via IP injection or saline, initiated at 3dpi and continued for 10 days. This ET-1 analog was used rather than BQ123 in order to avoid a second major surgery and additional rounds of anesthesia in our experimental mice.

Parasitemia (percentage of pRBCs) was assessed everyday by examination of tail blood smears stained with Giemsa. Weight, temperature, and RMCBS were also monitored daily for health status throughout the study. In some experiments, PbA-infected mice were allowed to manifest the signs of ECM, as previously described, then treated with Artemether (at a dose of 50mg/kg; kind gift from Dafra Pharma GmbH, Switzerland) dissolved in coconut oil and administered by i.p. injection, for a total of 5 days (Figure S4)[[12](#_ENREF_12)].

In a separate set of experiments PbN-infected mice were treated daily with either exogenous ET-1, dissolved in normal saline (7.5ug/mouse; Millipore), or with normal saline, administered by i.p. injection beginning at day 3 after infection.

Mice were sacrificed and intracardially perfused with ice-cold PBS. Brains were excised and stored at -80°C for analysis.

**Cognitive Tests.** During the object recognition test animals underwent a training and test trial, as previously described [[9](#_ENREF_9), [10](#_ENREF_10), [64](#_ENREF_64)]. This test is based off the inherent tendency of mice to preferentially explore novel objects [[9](#_ENREF_9), [10](#_ENREF_10), [64](#_ENREF_64)]. In the training trial mice were gently placed in an open field with two identical objects. Mice were allowed to habituate and object exploration of each object was recorded. Mice were then returned to their home cage for a retention interval of 60min. After a 60min retention interval mice were returned to the open field with the one familiar object and one novel object. Again exploration of each object was recorded.

Healthy mice preferentially explore novel objects. Data are represented as the amount of novel object exploration divided by the total time exploration of both objects. A preference score of 50% indicates chance performance, whereas a score greater than 55% indicates novel object preference. Adolescent mice (i.e. post-natal days 21-60) display a basal increase in impulsivity and risk-taking behavior when compared to adult mice; this includes increased novelty seeking [[67](#_ENREF_67), [68](#_ENREF_68)]. Since our mice are initially tested between 6-8 weeks, a novel object preference between 50-55% may simply reflect a basal increase in hyperactivity and exploratory behavior [[68-70](#_ENREF_68)], and not necessarily true preference. Based on our observations over many years, a 55% score has reliably been reflective of true novel object preference. Test objects were counterbalanced to prevent confounds of preferential exploration of an object. Examiners were blind to infection and treatment conditions of the mice.

**Cranial Window Surgery and Intravital Microscopy.** A closed cranial window was used as previously described [[7](#_ENREF_7)]. Briefly, mice were anesthetized, the scalp was then removed and a craniotomy of 3-4 mm in diameter was created, using a surgical drill. The exposed tissue was covered with a 5mm glass coverslip. Two weeks after surgery, a panoramic photograph of vessels was taken and a map of selected vessels was created. One micrometer was equivalent to 2.57 pixels; thus, vessel diameter in μm was calculated as measured pixels/2.57. Approximately 7-10 vessels were imaged in each animal at baseline and 6 dpi. Vessel diameters at 6 dpi were compared to baseline measurements. Data are represented as percent change in vessel diameter to baseline measurements.

Blood brain barrier (BBB) disruption was assessed as previously described [[65](#_ENREF_65)]. Briefly, experimental mice received a tail vein injection of Tracer-653 (Molecular Targeting Technologies, Inc.) diluted in PBS [[65](#_ENREF_65)]. Four hours later mice were anesthetized with ketamine and xylazine and placed inside an *in vivo* imaging system (IVIS) Kodak Image Station 4000MM PRO (Carestream Health) equipped with a CDD camera. In some experiments, mice were anesthetized, intracardially perfused and imaging was performed on excised brains. Acquired images were analyzed with Carestream MI 5.3.17476 Application Software.

**Histology.** Mice were anesthetized by isoflurane inhalation and perfused through the heart with saline. Brains were excised, fixed and embedded in paraffin. Brains were cut into 4μm sagittal sections and stained with hematoxylin-eosin.

Congestions score: Approximately 500 vessels were analyzed per group. Congestion scores were based on a scale of 0-4; 0 = No cells present within the vessel lumen; 1 = vessels with a minimal amount of cells, including RBCs, platelets, and adherent leukocytes, obstructing less than 10% of the vessel lumen; 2 = vessels with a moderate degree of cellular obstruction, with 10% to 50% occlusion of the vessel lumen; 3 = vessels with an extensive amount of cellular obstruction, with 50% to 90% occlusion of the vessel lumen; 4 = completely occluded vessel lumen.

**REFERENCES**

67. Laviola G, Macrı̀ S, Morley-Fletcher S, Adriani W. Risk-taking behavior in adolescent mice: psychobiological determinants and early epigenetic influence. Neuroscience & Biobehavioral Reviews. 2003;27(1):19-31.

68. Adriani W, Chiarotti F, Laviola G. Elevated novelty seeking and peculiar d-amphetamine sensitization in periadolescent mice compared with adult mice. Behav Neurosci. 1998;112(5):1152-66. PubMed PMID: 9829793.

69. Spear LP. The adolescent brain and age-related behavioral manifestations. Neurosci Biobehav Rev. 2000;24(4):417-63. PubMed PMID: 10817843.

70. Spear LP, Brake SC. Periadolescence: age-dependent behavior and psychopharmacological responsivity in rats. Dev Psychobiol. 1983;16(2):83-109. doi: 10.1002/dev.420160203. PubMed PMID: 6339302.
